# μBrain: An Event-Driven and Fully Synthesizable Architecture for Spiking Neural Networks

**DOI:** 10.3389/fnins.2021.664208

**Published:** 2021-05-19

**Authors:** Jan Stuijt, Manolis Sifalakis, Amirreza Yousefzadeh, Federico Corradi

**Affiliations:** Ultra-Low-Power Systems for Internet of Things (IoT), Stichting Interuniversitair Micro-Elektronica Centrum (IMEC) Nederland, Eindhoven, Netherlands

**Keywords:** spiking neural network, neuromorphic computing, radar signal processing, IoT, edge-AI

## Abstract

The development of brain-inspired neuromorphic computing architectures as a paradigm for Artificial Intelligence (AI) at the edge is a candidate solution that can meet strict energy and cost reduction constraints in the Internet of Things (IoT) application areas. Toward this goal, we present μBrain: the first digital yet fully event-driven without clock architecture, with co-located memory and processing capability that exploits event-based processing to reduce an always-on system's overall energy consumption (μW dynamic operation). The chip area in a 40 nm Complementary Metal Oxide Semiconductor (CMOS) digital technology is 2.82 mm^2^ including pads (without pads 1.42 mm^2^). This small area footprint enables μBrain integration in re-trainable sensor ICs to perform various signal processing tasks, such as data preprocessing, dimensionality reduction, feature selection, and application-specific inference. We present an instantiation of the μBrain architecture in a 40 nm CMOS digital chip and demonstrate its efficiency in a radar-based gesture classification with a power consumption of 70 μW and energy consumption of 340 nJ per classification. As a digital architecture, μBrain is fully synthesizable and lends to a fast development-to-deployment cycle in Application-Specific Integrated Circuits (ASIC). To the best of our knowledge, μBrain is the first tiny-scale digital, spike-based, fully parallel, non-Von-Neumann architecture (without schedules, clocks, nor state machines). For these reasons, μBrain is ultra-low-power and offers software-to-hardware fidelity. μBrain enables always-on neuromorphic computing in IoT sensor nodes that require running on battery power for years.

## 1. Introduction

Information processing in the brain has been a topic of active research for decades (Cappy, 2020). As a computing substrate, the brain structure is exciting from an engineering perspective. It is massively parallel, impressively low power, enables scalable operation, and memory and computation are multiplexed together in the same substrate. As a result of the study of the brain, research in neuromorphic computing has been trying to build brain-inspired models of information processing and respective hardware implementations thereof.

Unlike conventional computer architectures designed to perform exact calculations, a biological brain seems optimized for signal processing in the presence of noisy or incomplete inputs. It is very robust to damages and partial failures. As a result, neuromorphic computing offers an alternative for algorithms and compute architectures that perform (statistical) signal processing and neural processing tasks. Even though we are far from having understood the brain's functioning altogether, the study of its operation leads us to several important architectural features, which we can successfully and effectively adopt in silicon technology of computing machines.

Many of the brain's energy and compute efficiency features come from its asynchronous and event-driven operation (Yu and Yu, 2017), which promotes and simultaneously exploits sparse computations. In conventional processor/accelerator architectures where high-energy consumption is unavoidable, the focus is on maximizing efficiency (and speed) by increasing the number of operations possible per unit of energy consumed. By contrast, in neuromorphic architectures, sparsity exploitation results in skipping redundant operations, and efficiency is achieved by directly reducing both latency and energy consumption. Reducing operations translates to fewer computations and less power density (i.e., power per silicon area) in the neuromorphic processors. Besides, asynchronous event-driven processing allows for theoretically infinite scalability as every neuron can process its inputs independent of other neurons. It also lets the information flow as fast as possible, which results in a low latency response. It is not required to route a dynamic clock pulse to every neuron in a silicon implementation, as each neuron immediately evaluates its membrane potential against the threshold without the need for a global synchronization signal (a clock).

This paper introduces μBrain, a neuromorphic IC for ultra-low power (<100 μW) neural network processing for edge AI IoT applications. μBrain exploits low-cost digital technology, but unlike most other digital neuromorphic Integrated Circuits (ICs) (as shown in **Table 2**), it relies on local on-demand oscillators and a novel delay-cell to avoid the use of a global clock and it supports event-driven processing. μBrain, in the absence of input stimuli, only consumes leakage power while maintaining its internal state stored in the neuron's membrane potential, synaptic weights, and network dynamics. Furthermore, μBrain does not exploit separate memory blocks (either on -chip or off-chip memory), but memory and computation are co-localized in the IC area, avoiding the data access and energy overheads of distal memories of conventional Von-Neumann architectures.

The use of digital technology leverages synthesizability, and it provides reliability for use in various IoT applications. Besides, the high area efficiency of digital gates offered in advanced process nodes makes analog neurons less attractive.

The μBrain architecture is based on digital event-based spiking neurons organized in layers (recurrent topologies are also supported). Inputs and outputs are digital pulses (rate- or time-coded), whereas the synaptic weights are programmable and are stored on-chip with a customizable bit-width. Depending on the application requirements, the μBrain architecture can be customized during synthesis for bit precision, network topology (number of neurons in each layer, and number of layers), and connectivity. In contrast, neuron parameters and synaptic weights are runtime programmable.

The niche of μBrain in the landscape of neuromorphic processors and accelerators is ultra-low-power (e.g., hundreds of μW) lightweight machine-learning data processing near- or in-sensor (and by “in-sensor” we mean integration at the IC level). Example target deployments include radar signal classification, biomedical signal analysis on wearable devices, low-dimensional image classification deployed on luminaires, audio analysis and tactile sensing analysis in thin-film electronics, data processing on ingestible sensors, and many other IoT applications.

### 1.1. Background and Related Literature

Neuromorphic compute accelerator ICs leverage Spiking Neural Network (SNN) processing, using stateful neuron models that exchange information in the form of sparse asynchronous events (spikes). State-of-the-art implementations are based on analog, digital, or hybrid mixed-signal silicon technology (such as Schemmel et al., 2010; Qiao et al., 2015; Furber, 2016; Neckar et al., 2018), often in combination with “exotic” non-volatile memories (NVM) (Zhang et al., 2018), or photonic technology (Prucnal and Shastri, 2017), or spintronic devices (Grollier et al., 2020). This broad range of options accounts for varying degrees of emulation of the real brain structures, integration, and features.

Analog neuromorphic ICs resemble the biological neural cells more than digital ICs (Indiveri et al., 2011). They model potassium and sodium channels and N-methyl-D-aspartate (NMDA) receptors with their intricate dynamics. Yet, they suffer from variability, high design cost, low flexibility, and low neuron density. When implemented in conventional silicon technology, neurons store their membrane potentials (neuron states) in a leaky capacitor, which costs a large area, and analog synaptic circuits mimic adaptation and learning with programmable synaptic weights with low digital resolution (Bartolozzi and Indiveri, 2007). Alternatively, a dense Resistive Random Access Memory (ReRAM) crossbar may be used to build the synaptic connections between neurons (Liu et al., 2015). In ReRAM crossbars, the bit cell's resistance is the programmable synaptic weight that connects a presynaptic with a post-synaptic neuron. Due to process variations, the analog chips are not exactly reproducible and are vulnerable to temperature changes. In theory, it is possible to overcome the variations by using an adaptive self-learning neuron model and efficient on-chip adaptivity/learning mechanism to compensate for the variations and noise (Kuzum et al., 2012). However, such mechanisms make the neuron more complex. Their performance is not yet sufficiently reliable to enable the use of such technology in critical applications (e.g., health care, automotive, safety). The analog approach is not suitable for our work as μBrain targets inference only, IoT use cases, and easy and affordable reproducibility and integrations with other ICs (e.g., sensors) leveraging in-sensor processing.

By contrast to analog circuits, digital ICs rely on logic gates to emulate neurons and synapses and dense memory to store neuron state and synaptic weights (Frenkel et al., 2018). This approach's motivation is to make a synthesizable architecture integrated quickly in a System On a Chip (SoC) and results in a low-cost implementation. In theory, due to using logic gates, the required area in this approach can be higher than in analog chips. However, it is easier to use state-of-the-art technology nodes (like 7 nm and below) for digital, which offers much better density at reasonable power consumption. One disadvantage of digitally designed chips is the implementation of membrane potential leakage as an additional periodic operation. This disadvantage is not so relevant if the frequency is low enough, i.e., in the same order as the input spike rates. Besides this, since commercial electronic design automation (EDA) tools are optimized for synchronous deployments, it is not straightforward to implement fully event-driven implementations.

Likewise, in μBrain, we followed a fully digital approach. However, our leakage mechanism is event-based and, therefore, does not necessarily need to be periodic. Additionally, we have designed a lightweight local oscillator (a delay cell) that can drive self-timed digital blocks (similar to Davies et al., 2018) to overcome the lack of support in Electronic Design Automation (EDA) tools.

At the intersection of these two approaches, mixed analog and digital neuromorphic ICs may combine analog circuit networks with a digital readout layer (Corradi et al., 2019) or an analog ReRAM crossbar for synaptic connections with digitally implemented neurons (Ni et al., 2017). In this case, at the interfacing between the analog and digital circuit, analog signals are discretized using an analog to digital converter. As activations in SNNs are binary (no multiplication is required), this method's main advantage is the possibility to store multiple bits in one memory cell. Additionally, bio-inspired learning algorithms can be implemented using resistive memory cells' physical characteristics and can facilitate on-chip learning. Even though μBrain is compatible with non-volatile memory technologies as a replacement of the distributed memory (digital flip-flops) for synaptic weights, we ruled out the analog option for the reasons mentioned before.

As electrons' speed is much faster than ions, a silicon neuron can process spikes some orders of magnitude faster than its real-time biological equivalent (nanoseconds switching on/off time for transistors, vs. milliseconds neuronal and synaptic time constant). This fact has motivated neuromorphic digital IC engineers to implement time-multiplexed digital neuromorphic chips (Davies et al., 2018, Merolla et al., 2011). In digital implementations, it is possible to separate the processing part and the memory. For example, one physical neuron core can emulate many (virtual) neurons and one physical link to emulate many (virtual) synaptic connections. Time-multiplexing methods employ fast computations and constantly shuffle neuron's membrane potential from/to neuron memory and their synaptic weights from/to synaptic memory. Furthermore, such an architecture may host multi-neuron cores, each assigned the emulation of a group of neurons, e.g., a layer, which can exchange spikes asynchronously in a packet-switched form through a network-on-chip (NoC); and based on the Address-Event Representation (AER) of spikes in packets. The advantage of the time-multiplexing approach is a higher neuron and synapse density compared to the previous approaches and leveraging of more complex neuron models [or even programmable (Painkras et al., 2013)] at the cost of increased memory access and complex data-shuffling primitives. Time-multiplexing may be disadvantageous for ultra-low-power designs as it requires additional control circuitry, increasing power consumption to manage the core's coherence. Also, contra to biological neurons, the distance between memory and compute cores increases the power consumption. As events inside each core are processed serially, at peak activity times, processing latency also increases or is not guaranteed and may result in event drop out (depending on the depth and occupancy of event queues). Finally, packetization and explicit addressing of events (as in AER protocols) increase communication overhead (power consumption) due to the additional address processing and routing and memory requirements for queueing events in transit (events are not a binary pulse or a direct signal anymore). In the μBrain architecture, we do not time-multiplex the processing of multiple neurons in a core (rather, each core is assigned exclusively to one neuron) because for the size of networks we are considering, the total silicon area of neurons is negligible compared to the total area of synapse memory. In addition, a packet-based event addressing is not required internally among neurons, but we have opted for AER communication at the chip interface with the outside world for ease of integration with existing neuromorphic sensory systems.

The μBrain area is memory dominated, which is not a good characteristic. However, μBrain requires distributed memories and motivates the search of alternative memory technologies to Static Random Access Memory technologies. Many novel memory technologies are currently being investigated as candidate solutions for neuromorphic technologies, such as Phase Change Memories (PCM) (Nandakumar et al., 2018), Resistance switching memory (RRAM) (Indiveri et al., 2013), Electrochemical Metalization Memories (ECM) (Hao et al., 2021). For this reason, our architecture is not focusing on the memory aspect, as it could soon be replaced with some of the novel technologies.

## 2. Materials and Methods

### 2.1. Event-Based Architecture

**Figure 1:**
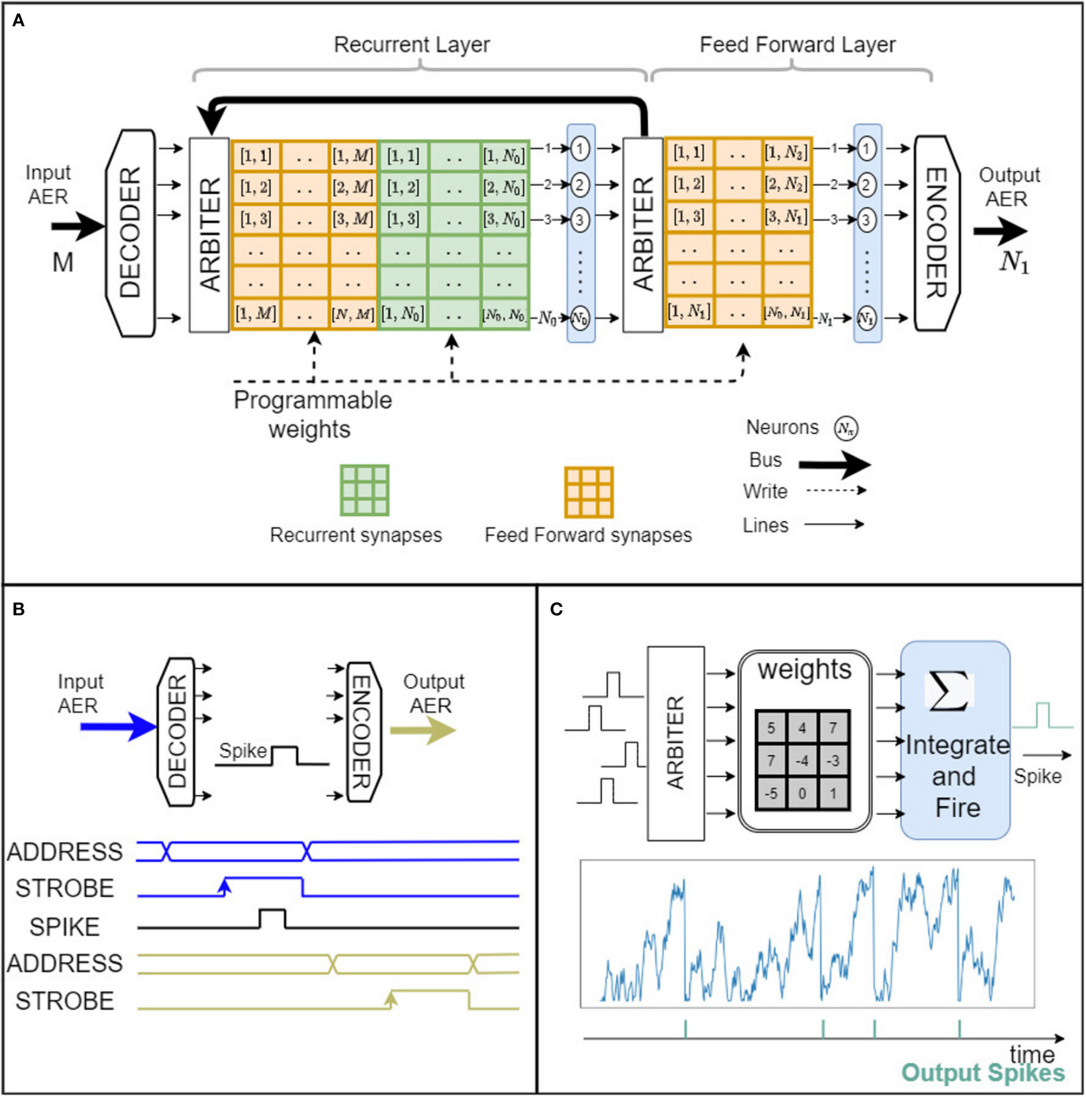
μBrain event-driven architecture. **(A)** The digital architecture is organized in layers. Each layer consists of an arbiter, a weight memory matrix for forward and recurrent connections, and a set of IF neurons. The architecture can be synthesized for an arbitrary number of neurons, weight bit width resolution, and synaptic memory size *M*, *N*_*x*_ – where *M*, is the number of inputs and *N*_*x*_ is the number of neurons in layer indexed by *x*. **(B)** Input/Output address event representation signals and timing. **(C)** Simplified schematic of a digital spiking neuron. Input spikes arriving at random times select corresponding weights, which in turn are added (or subtracted) by an accumulator. Each time the accumulator overflows, the neuron's circuit emits an output spike on the axon output. The graph below shows the time progress of the accumulator value representing the neuron's membrane potential. Output spikes are shown below the neuron's membrane potential.

An overview of the main building blocks of the μBrain architecture and their interactions is provided in Figure 1A. Event-based integrate-and-fire (IF) neurons are arranged in a fully parallel topology of layered populations, which means that each neuron is physically implemented in silicon (not time-multiplexed). Within each layer, there may exist lateral synaptic connections (that can leverage recurrent connectivity). Every neuron independently (no global clock) accumulates weighted incoming synaptic spikes and emits a spike itself when the neuron's accumulator overflows. Input spikes trigger the membrane voltage integration, with immediate threshold evaluation, resulting in distributed granular activations. As input pulses arrive asynchronously before a neuron layer, an event arbiter resolves any ordering conflicts if spikes arrive simultaneously. Synaptic weights have a fixed bit-width (determined at synthesis) representing 2's complement integer quantized values, in the range [−2^*W*−1^ − 1, +2^*W*−1^ − 1], where W represents the number of bits. For a given bit-width, the range of quantized weight values can be linearly or logarithmically arranged (the latter case has been taken into account since precision is often more critical for smaller weight values).

Note that while the neuron implements an Integrate-and-Fire (IF) neuron model (see Figure 1C), a Leaky Integrate and Fire (LIF) model can also be facilitated by using one of the neuron inputs to provide a periodic leakage signal. This will necessitate an external clocked input (see Figure 1C).

### 2.2. Input/Output Interface

Input and output spikes are transmitted to/from μBrain using a simple communication protocol based on the Address Event Representation (AER). Unlike other common neuromorphic AER systems (Boahen, 2000), which rely on a handshake mechanism, μBrain uses only a strobe signal whose rising edge informs when the address data are ready to be parsed (Figure 1B). The strobe is then kept high for a few ns to indicate a time duration that the address data remain valid and a spike is propagated throughout the network.

The AER representation allows seamless interfacing with event-based sensors like the silicon retina (Lichtsteiner et al., 2008) and silicon cochlea (Liu et al., 2010), and microcontrollers to perform further downstream spike-based signal analysis (classification, regression, etc.).

### 2.3. Spike Arbiter

**Figure 2:**
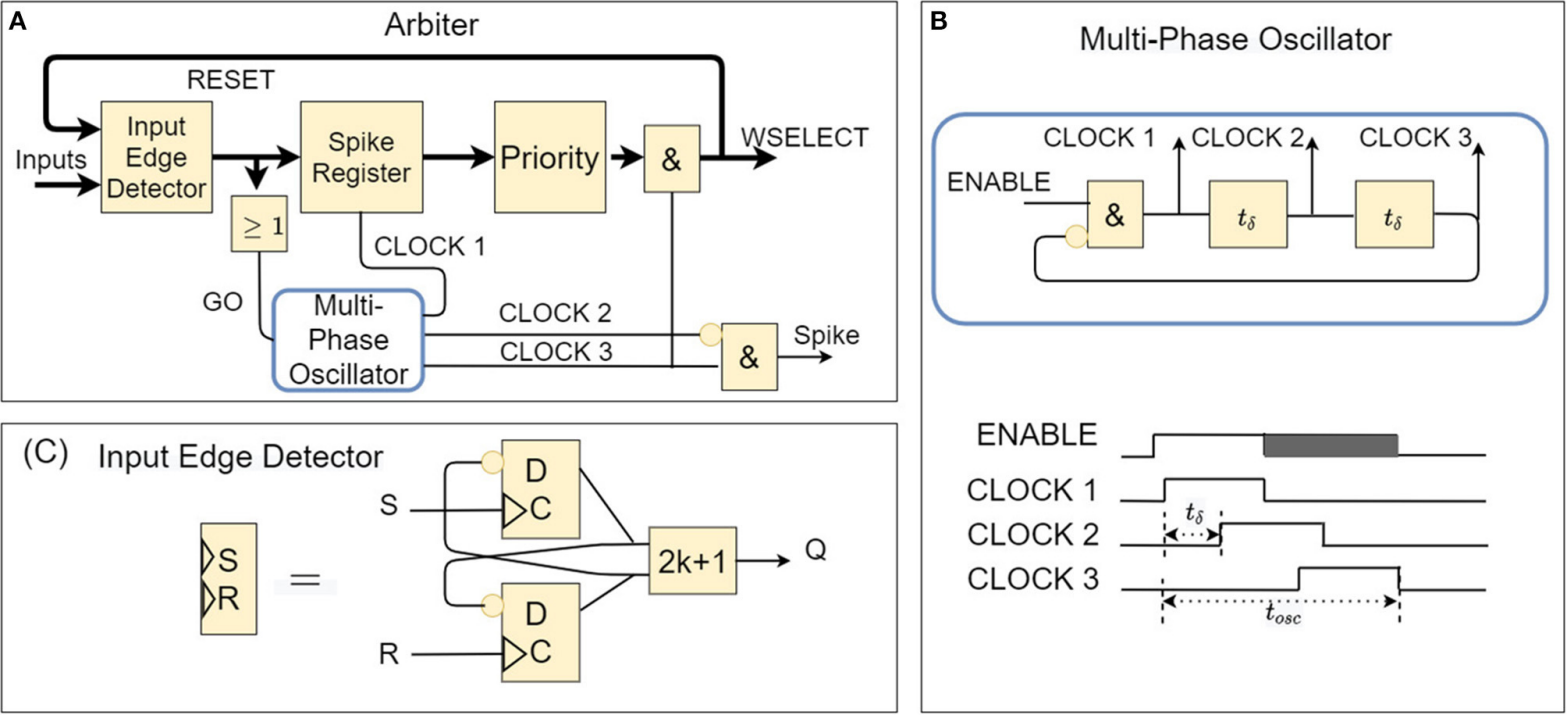
**(A)** Logic block diagram of spike arbiter (thick lines represent many parallel signals). **(B)** Logic block diagram of the local oscillator and the timing of the self-generated clock pulses. **(C)** Logic block diagram of the input edge detector (edge-triggered) implemented through an S/R circuit. It is parameterizable with a parameter *H* representing the number of states (so it can remember *H-1* spikes). Here we show the case in which (H = 2), i.e., the most straightforward configuration.

The spike arbiter before each layer of neurons (shown in Figure 2A) detects the presence of at least one input spike and dispatches it to the recipient layer neurons. When more than one spikes arrive simultaneously, the spike arbiter takes care of ordering and spacing them in time^1^. The arbitrations delays are in the order of ns, while the incoming spikes arrive with a spacing in the order of μs, or even *ms* (input frequencies range from Hz to hundreds of kHz).

This functionality is implemented as follows (see Figure 2A). Incoming spikes trigger an Input Edge Detector (implemented as shown in Figure 2C) and are immediately propagated to a spike register before the Priority-Encoder. A round-robin or linear polling algorithm generates a 1-hot encoded mask, which gets applied to the spike register contents to select a single spike for propagation. Suppose there has been registered more than one simultaneous spike in the spike register. In that case, the difference between the spike register contents and the masked output (i.e., remaining spikes) are fed back to the Input Edge Detector for subsequent recursive processing (until all spikes are consumed one-by-one by the Priority-Encoder). The spikes that come out of the arbiter (see Figure 1C) activate (index) parts of the post-synaptic weight memory to select weight values from the fan-out synapses into the respective neurons' accumulators; to incrementally implement a weighted spike integration at each downstream IF neuron.

Upon the arrival of incoming spikes and throughout their consumption, the arbiter circuit becomes on-demand self-clocked by means of a multi-phase single-cycle oscillator and a special delay-cell circuit (explained next).

### 2.4. The Multi-Phase-Oscillator and Delay Cell

In the absence of a global system-clock, the Multi-Phase-Oscillator (Figure 2B) is an on-demand activated local clocking circuit at the heart of the arbiter that warrants correct pacing of its phases for ordered propagation of spikes among neurons and across layers; and in this sense, it is the key component for the event-driven operation of μBrain. The primary sophistication that enables this functionality is a delay-cell (within the multi-phase-oscillator).

Whenever (at least) one spike is latched in the arbiter and propagated to the priority encoder, it sets off one oscillation cycle in the multi-phase-oscillator, which by means of the delay cell gets delivered in sequence at different places of the arbiter to activate, temporarily only, first the loading of the spike register in the priority encoder, then trigger the 1-hot masking/selection of a spike, and finally activate the synaptic memory selector. Its operation is depicted in Figure 2C.

**Figure 3:**
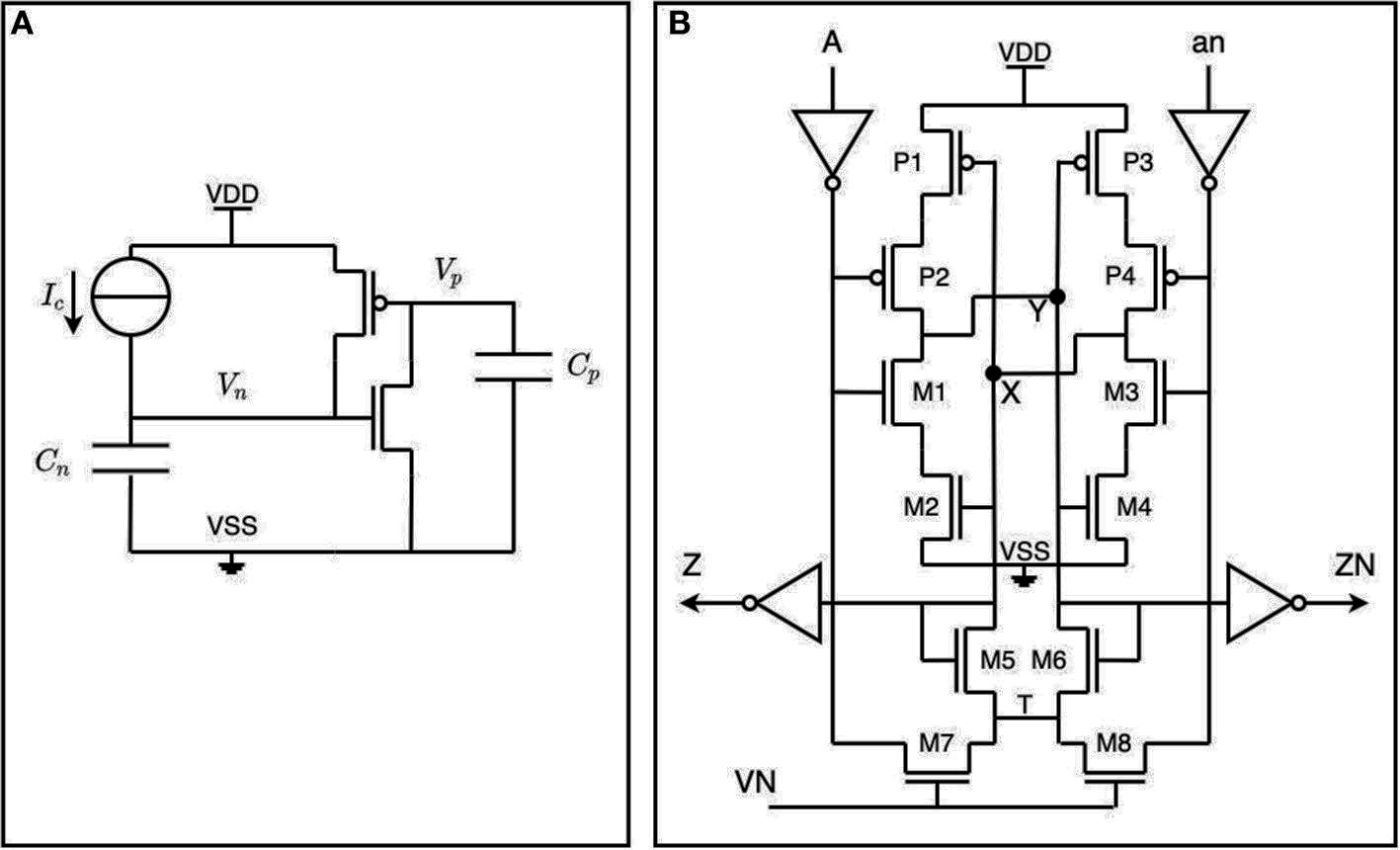
Schematic design of the delay cell. **(A)** A CMOS thyristor is a combination of a PMOS and an NMOS transistor, in which the drain of the PMOS is connected to the gate of the NMOS. **(B)** Two cross-coupled CMOS thyristors implementing a delay-cell.

The delay cell's generated delays are fixed and take into account the maximum input spike frequency, various integration technology variation parameters, and the overall timing constraints of the circuit during synthesis/place-and-route of the IP. The current prototype operates in a few ns (we used 100ns to have a safe margin). This is a substantially large delay given that in standard CMOS technology timing circuits are generally energy-consuming. It is, however, possible to make considerable delays (hundreds of ns to hundreds of μs) without sacrificing power dissipation using CMOS thyristors (Zhang et al., 2004). Our design uses two thyristors in a cross-coupled configuration (see the schematic of Figure 3B), in which the current in the delay cell is limited with a near-threshold bias voltage. The final layout of this cell is compact and, in our design, requires 3.0 μm^2^. The delay must be within safe margins while its actual value does not need to be precisely tuned. In the face of these challenges, the delay cell's custom design plays a crucial role in μBrain's low power consumption.

The delay generation is explained as follows: assume that *V*_*n*_ = 0 and *Vp* = *Vdd* such that both transistors are off (see Figure 3A). Then, because of the current source *I*_*c*_, *V*_*n*_ goes up linearly until *V*_*n*_ = *V*_*tn*_ during a time *t*_*d*1_ when the NMOS transistor starts to conduct:

(1) $$\begin{array}{l} t_{d1}=\frac{C_{n}V_{tn}}{I_{c}} \end{array}$$

Voltage *V*_*n*_ keeps going up linearly:

(2) $$\begin{array}{l} V_{n}\left(t\right)-V_{n}=\frac{I_{c}}{C_{n}}t \end{array}$$

*V*_*p*_ goes down until *Vdd* − *V*_*tp*_ during a time *t*_*d*2_ when the PMOS transistor starts to conduct:

(3) $$\begin{array}{l} I_{dn}=\frac{\beta_{n}}{2}{\left(V_{n}-V_{tn}\right)}^{2}=\frac{\beta_{n}}{2}{\left(\frac{I_{c}}{C_{n}}\right)}^{2} \end{array}$$

The charge on capacitor *C*_*p*_ is simply the integral in the *t*_*d*2_ time interval, as:

(4) $$\begin{array}{l} \int_{0}^{t_{d2}}I_{n}dt=C_{p}V_{tp} \end{array}$$

Which means that *t*_*d*2_ is:

(5) $$\begin{array}{l} t_{d2}=\sqrt[{3}]{\frac{6C_{n}^{2}C_{p}}{\beta_{n}I_{c}^{2}}} \end{array}$$

After, the voltages quickly move to *V*_*n*_ = *V*_*dd*_ and *V*_*p*_ = 0 Finally the total delay time *t*_*d*_ results in:

(6) $$\begin{array}{l} t_{d}=t_{d1}+t_{d2}=\left(\frac{V_{tn}}{I_{c}}+\sqrt[{3}]{\frac{6V_{tp}}{\beta_{n}I_{c}^{2}}}\right)C_{L} \end{array}$$

Where *C*_*L*_ = *C*_*p*_ = *C*_*n*_.

The current in the CMOS delay cell (Figure 3B) is limited with a near-threshold bias voltage on node *V*_*N*_. The delay between node *A* and *X* tracks with process variations, voltage, and temperature (PVT).

## 3. Results

This section presents an evaluation of an instantiation of μBrain's IP in a 40 nm technology node. For reference comparison of μBrain with other tiny spiking neural network processors, we perform the standard benchmark of handwritten digits recognition (MNIST). We also showcase the capabilities of μBrain while performing a radar-based hand gesture classification task.

### 3.1. μBrain's ASIC Prototype

**Figure 4:**
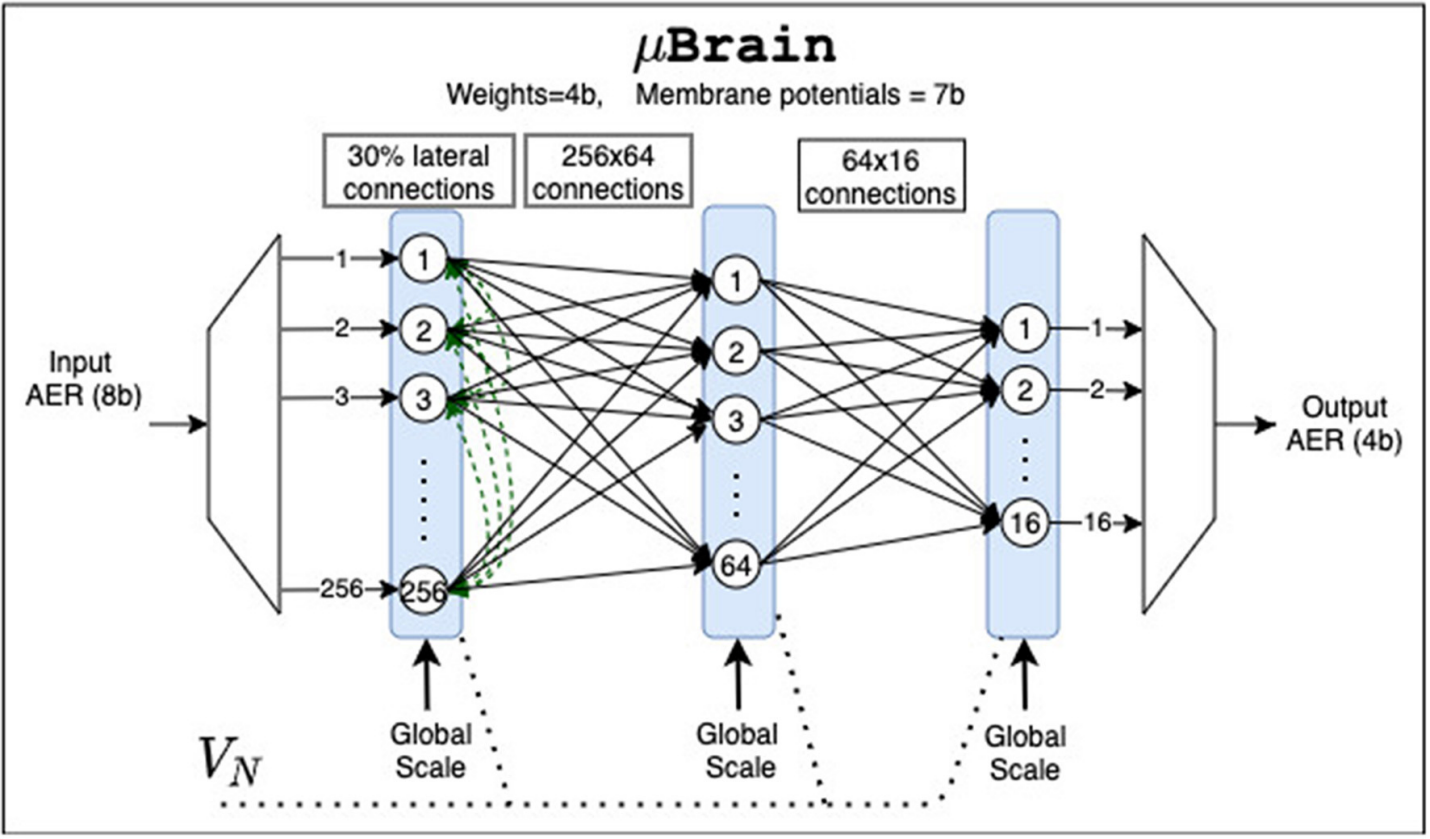
μBrain's ASIC instantiation for the experiments in this paper consists of three layers: a recurrent layer of 256 neurons with circa 30% lateral connectivity and two fully connected layers counting 64 and 16 neurons, respectively. *V*_*N*_ is the global near-threshold bias voltage used to tune the delay cells. The global scale inputs are digital inputs used to set to scale within a layer the synaptic weights.

We have produced a prototype implementation (see Figure 4) consisting of 336 neurons organized in a Recurrent Fully Connect (RFC) layer of 256 neurons, followed by two Fully Connected (FC) layers of 64 and 16 neurons, respectively. The synaptic weights' resolution in all layers has been fixed to 4 bits, representing discrete values from −7 to +7. The weights are runtime re-programmable in local flip-flops, organized via a shift register circuit. The RFC layer has a random connectivity pattern of about 30%, allowing savings in weight memory and using it as a reservoir. After the RFC layer, two FC-connected layers can serve as a second shallow network or can act as a readout classification network. The RFC has 19,878 weight registers (synapses), and the FC has 17,488, which is a total of 37,366. This adds up to 149,464 distributed memory bits (18.2 kB). Both RFC and FC have a global-scale input. When active, the synaptic weights get scaled by a factor of 8 before being accumulated in the neurons. The scaling option sets the threshold to 8 instead of 64. The neuron accumulators' size is 7 bits and can effectively store only positive values from 0 to 63. A neuron will generate an output spike when its accumulator value (i.e., “membrane voltage”) overflows. In that case, the accumulator content will not be reset but rather wrapped around. The accumulator's wrapping implies that the neurons reset to the overflow amount after emitting a spike. If a spike causes an underflow, the neuron accumulator is kept to zero. Each FC neuron has a bias input with a corresponding synaptic weight value. The global bias input emulates linear membrane leakage. The reset of the membrane potential at the overflow amount enables to map the behavior of the μBrain neurons to the Rectified Linear Units (ReLU) activations in a mean-rate approximation (to ease ANN to SNN conversion).

**Figure 5:**
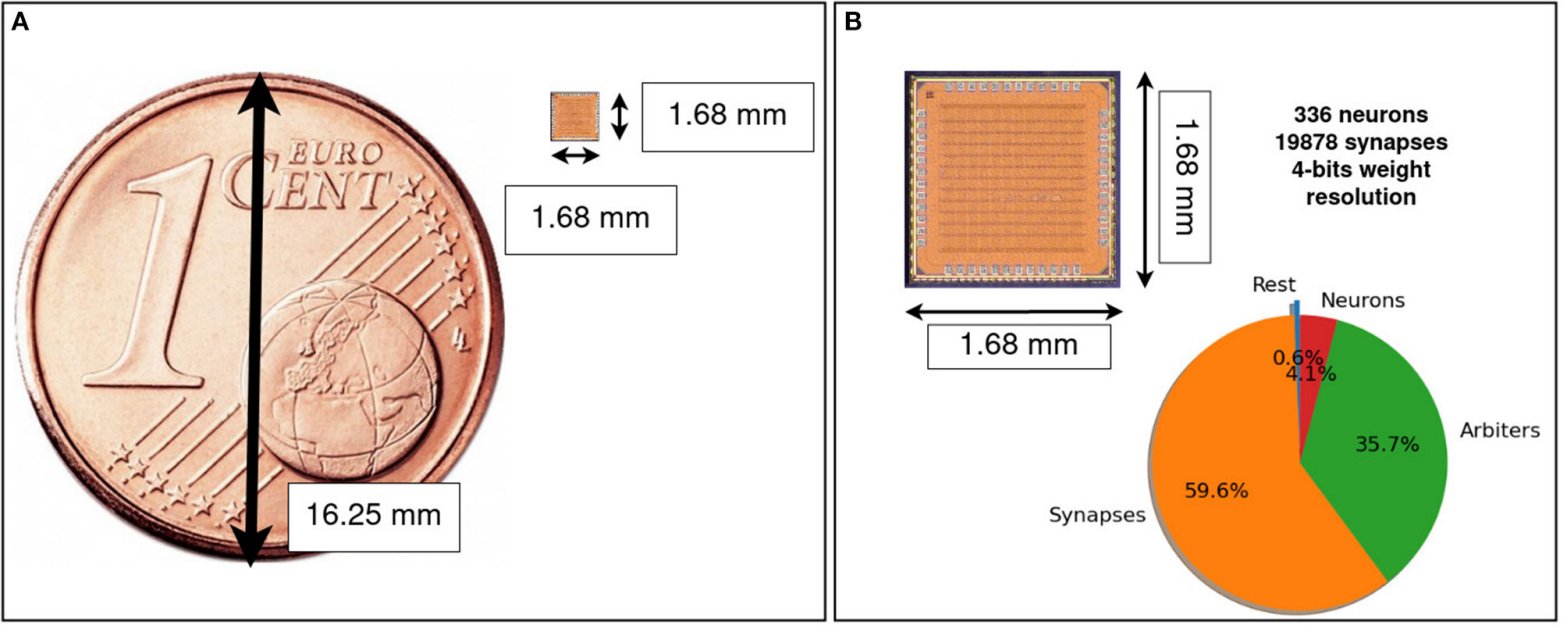
μBrain's micro-graph, the IC is implemented in 40 nm TSMC technology with an I/O voltage of 2.5 V and a core voltage of 1.1 V. **(A)** Micro-graph picture compared with a Euro cent coin. IC area is 2.82 mm^2^ (including pads). **(B)** Area breakdown: 59.6% flip-flops for synaptic weights and tri-state weight selectors (synapses), 35.7% spike arbiters, 4.1% neuron accumulators, and 0.6% remaining routing logic. Memory is completely distributed over the area (no Von-Neumann bottleneck).

μBrain layout area is 2.82 mm^2^, we used the 40 nm TSMC technology with I/O voltage of 2.5 V, and a core voltage 1.1 V. A micro-graph picture of the prototype device is shown in Figure 5.

### 3.2. Handwritten Digits Classification With μBrain

μBrain is designed for inference only, and training spiking neural networks can be done off-line with various techniques (Rueckauer et al., 2017; Neftci et al., 2019; Sengupta et al., 2019). μBrain is compatible with both spike-time and mean-rate coding schemes. As a proof of concept, we tested the μBrain prototype with a mean rate approach in which we converted a pre-trained Artificial Neural Network (ANN) into a spiking neural network (as first introduced by Pérez-Carrasco et al., 2013). This choice has been dictated by the static nature of the MNIST images and the simplicity of training and testing offered by the standard deep-learning frameworks [e.g., Tensorflow (Shukla and Fricklas, 2018)]. For these reasons, we have also exploited a feed-forward ANN network without relying on recurrent lateral connections. We trained a fully connected network of Rectified Linear Units (ReLU) with 256 inputs, 64 hidden, and 10 output units, respectively, and no biases. Since our instantiation of μBrain has only 256 inputs, we reduced the MNIST input images to 16 × 16 pixels. Pixel grayscale values are mapped into firing rates for the first layer of 256 neurons. The grayscale values [0, 255] are linearly mapped in the arbitrary selected frequency range [100, 655 kHz].

After training, the ANN activation values are encoded in the spiking neurons through their mean rate activations^2^. The weight values transferred from the trained ANN model to the SNN remain the same but are quantized and scaled to fit the limited 4-bit precision in the μBrain instance (i.e., the range [−1, 1] maps to the integer range [−7, +7]). The network's output is read out using a single measure of Inter Spike Interval (ISI). The output neuron that has the shortest ISI is considered the correct output class, and the network can proceed to compute the following input.

**Figure 6:**
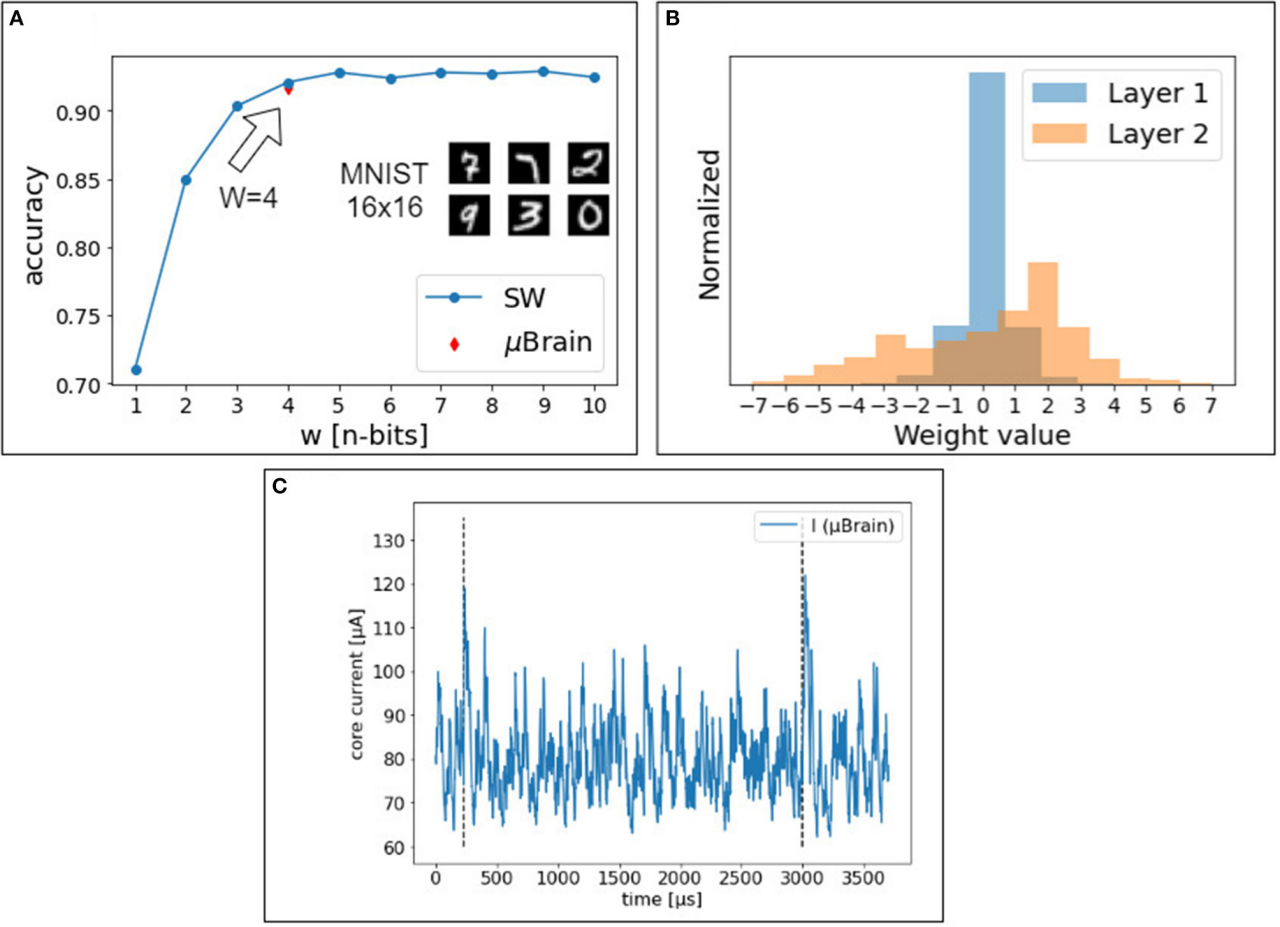
**(A)** Accuracy achieved in simulation and on the μBrain device, with a two-layer fully connected neural network. The red mark shows the accuracy achieved with the μBrain device. **(B)** Quantized weight distribution for the two layers of the shallow network. **(C)** The blue line shows the current measurement on the chip during handwritten digits classification (MNIST). The network is reset after two consecutive spikes are emitted by any output neurons (peak current reflects the reset). Vertical dashed lines indicate a single-digit classification. The mean current consumption for this digit is < *I* ≥ 88μ*A*, and it varies among test samples. To classify this digit, it takes 2,769 μs.

Figures 6A,B show the impact of weight quantization. The software simulation of the spiking neural network closely matches the hardware measurements. With <4 bit weights, the accuracy decreases significantly. The accuracy in the classification of the 10,000 digits in the MNIST test set (16 × 16 pixels) is consistently 91.7% (92% in the software trained model), with an average energy per prediction of 308 nJ. This performance is consistent with the literature (for the quantization scheme and size of the network used, as reported in **Table 2**).

### 3.3. Radar-Based Hand Gesture Classification With μBrain

Unlike vision-based imaging sensors, radar imaging systems directly capture motion profiles and temporal variations in the environment through active probing and intercepting the back-scattered power. Here, we applied machine learning to classify these motion patterns as previously proposed in Lien et al. (2016). To use our μBrain prototype in a radar signal classification use case, we converted the traditional micro-Doppler maps into tiny binary images that have been interpreted as spiking inputs for the μBrain device. These binary images indicate which of the 256 input neurons receive spiking inputs, just as in the case of MNIST. Binary images achieve comparable accuracy as grayscale input images, with no statistical difference. This motivates the use of micro-Doppler features as good features for gesture recognition. In contrast to camera-based vision, radar micro-Doppler can provide compressed outputs (sparse FFT coefficients) for faster inference while being robust in low-visibility conditions (e.g., in dark environments).

#### 3.3.1. Event-Based Frequency-Modulated Continuous-Wave (FMCW) Radar Sensor

For proof of concept experimentation, we used a low-power, low-resolution, 8 GHz Ultrawide-Band (UWB) Frequency Modulated Continuous Wave (FMCW) radar from Liu et al. (2019). The low range-resolution (<20 cm) and use of UWB technology in this radar make it a very low-power consumption sensor (20 mW), yet still very effective for various IoT applications, such as vital sign detection (Liu et al., 2019; Mercuri et al., 2019).

**Figure 7:**
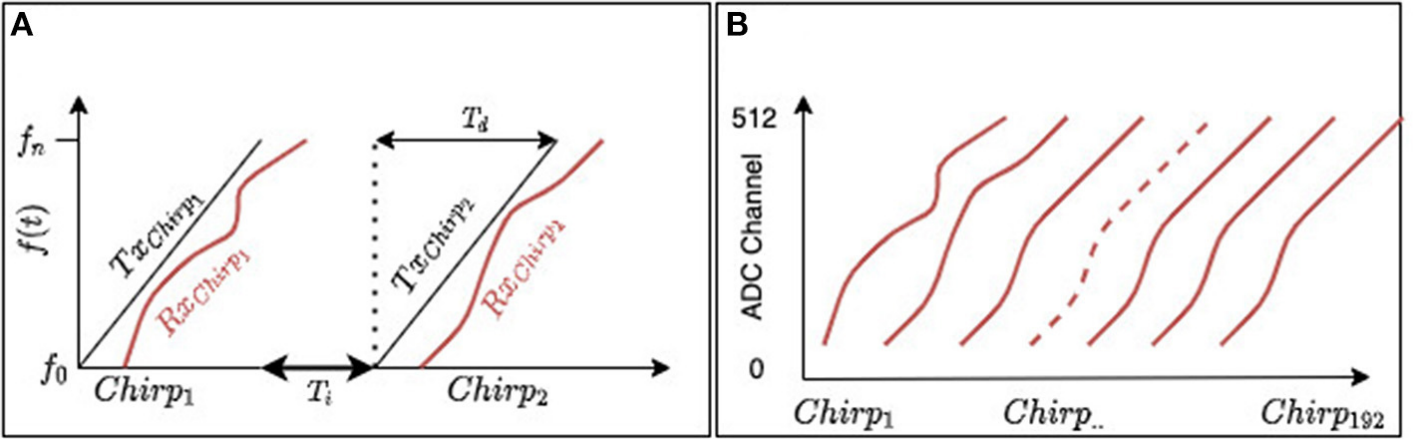
FMCW SISO radar signal illustration. **(A)** A transmitter antenna transmits a signal of linearly increasing frequency starting at *f*_0_ until *f*_*n*_. A receiving antenna captures back-scattered signal from the environment. *T*_*d*_ represent chirp duration, while *T*_*i*_ is the PRI (time interval) between chirps. **(B)** A radar frame is a collection of 192 consecutive chirp receptions.

FMCW radars transmit a continuous wave with linearly ramping up and/or down frequencies (chirp), starting from a frequency *f*_0_ up to frequency *f*_*n*_. Figure 7 shows a measurement of the back-scattered power. Here, we only state that the 8 GHz radar has a range resolution of about 30 cm, making it challenging to detect single finger movements, but enough to detect whole hand gestures' temporal trajectory. The bandwidth of a radar is defined as the frequency interval *B*_*w*_ = *f*_*n*_ − *f*_0_. This frequency interval defines the range resolution according to *res* = *c*/2*Bw*, in which *c* is the speed of light.

**Figure 8:**
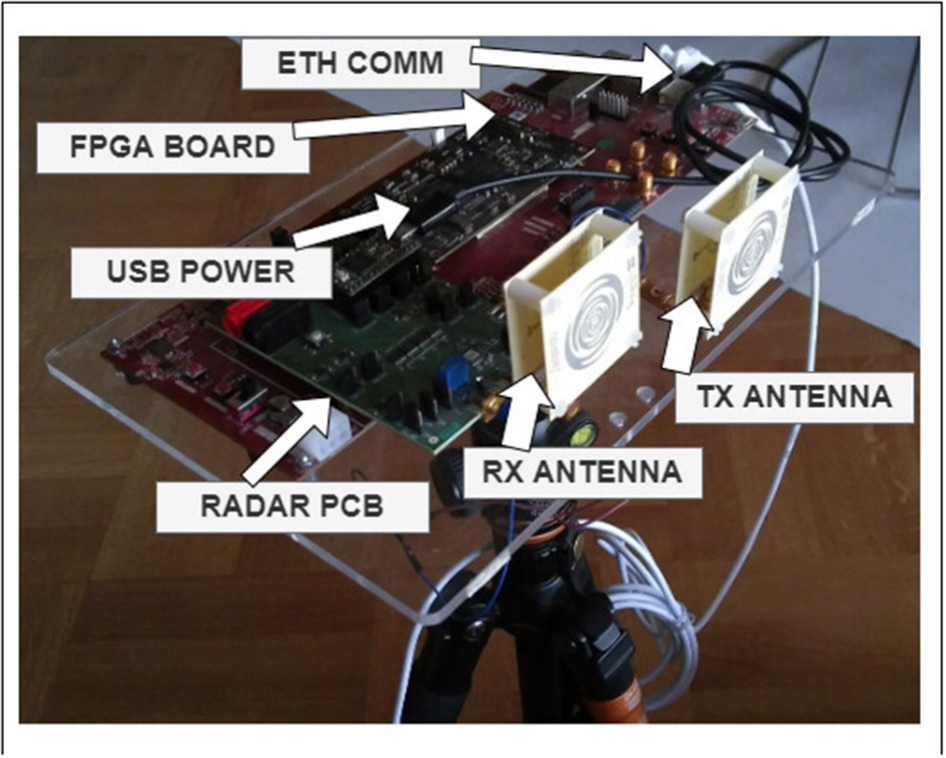
The lab prototype test platform on which the 8 GHz UWB FMCW radar IoT sensor IC is mounted for collecting data and carrying out measurements for vital-sign monitoring, activity classification, and other indoor applications.

A photo of the lab prototype platform on which the radar sensor IC is mounted is provided in Figure 8. This serves as a test platform for the pre-fabrication of a miniaturized IoT sensor for vital-sign monitoring, activity classification, and other indoor applications. In this prototype, the bulkiest part is an SoC platform, where backend logic (time-and-frequency domain) and communication is implemented and tested on a Field Programmable-Gate Array (FPGA) and embedded Linux processor. A Unix socket interface is used to communicate the spike event data to μBrain. The overarching objective is that the whole FPGA SoC will be obsolete and μBrain will be ultimately packaged in the same IC with the radar sensor. We refer the reader to Liu et al. (2019) for detailed circuits and operational range descriptions.

#### 3.3.2. Radar-Based Hand Gesture Classification in μBrain

**Figure 9:**
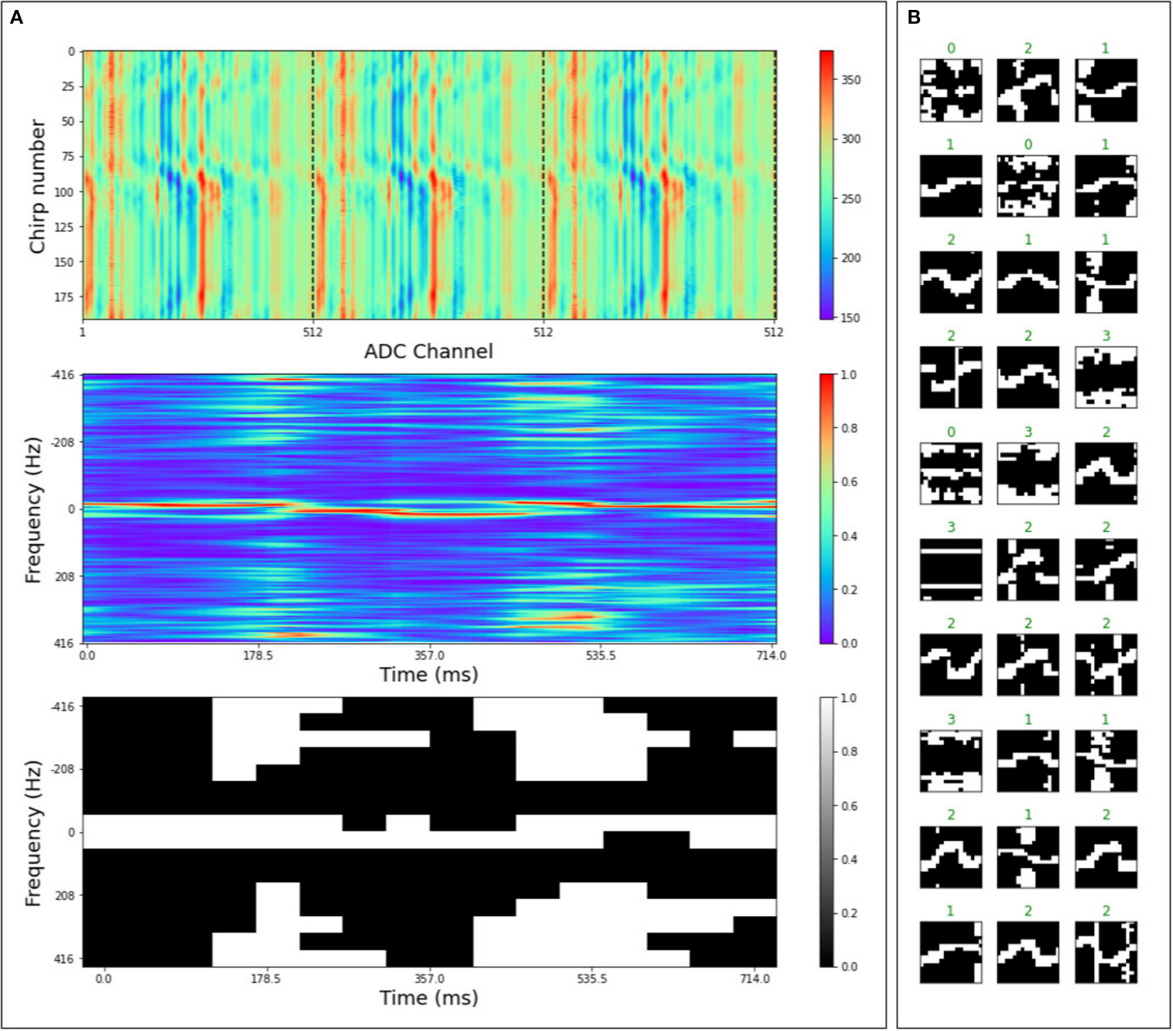
**(A)** Shows the preprocessing of the radar signal for 3 frames of raw ADC data (**A** top), to a micro-Doppler map (**A** middle), to a thresholded, scaled (16 × 16), and binarized version of the micro-Doppler map (**A** bottom). The binary image gets converted into a spike stream for the μBrain chip. **(B)** Shows examples from the preprocessed radar gesture dataset in which the label at the top associates to its respective gesture as 0: hello, 1: toward, 2: horizontal, 3: background.

With the aforementioned radar setup, we collected a hand-gesture dataset containing four dynamic gestures from five subjects. Data recordings include the subject standing at a distance of 2 m from the antennas (RX and TX). The gestures consist of swinging the right or left arm in the horizontal direction (horizontal), waving with the right or left hand by keeping the palm facing out (hello), moving the hand with the palm facing out radially toward and away from the radar (toward) and finally we recorded background activity in which none of the above gestures appeared in a static background (background). The radar system streams out chirp frames (collections of a fixed number of received chirp signal returns; as a 2d-matrix of time-domain data). In our setup, we collect 192 chirps in a single frame, while the number of ADC samples per chirp is 512. The ADC resolution is 10 bits. The time interval between emitted chirps has been set to *T*_*i*_ = 1.2 ms while the chirp duration is *T*_*d*_ = 41μ*s*; therefore, a frame consists of 238 ms of recordings. Figure 9 (top left) shows three successive frames divided by a vertical dashed line. The second figure from the top left in Figure 9 shows a micro-Doppler map obtained by processing three frames of radar signal (Chen et al., 2014) (computed as described in Supplementary Material). The micro-Doppler maps show the distribution of reflected energy over velocity, at a fixed distance, as a slow-time function. These maps thus provide rich information of the gesture dynamics over time. We converted the micro-Doppler maps into binary images, which serve as spike inputs, to directly interface the radar system with spiking neural networks in μBrain. In this conversion we apply a dynamic threshold on the micro-Doppler map, the threshold on the micro-Doppler map has been set to *Thr* = μ+*s*·σ, in which μ is the mean of the micro-Doppler map as $\mu =\frac{1}{n}\overset{n}{\underset{i=1}{\sum }}P_{i}$, σ is the standard deviation, and *s* a scaling factor (*s* = 0.15). The scaling factor is a hyper-parameter, serving as a crude noise filter by means of quantizing, and its optimal value is determined through grid search. After thresholding, the pixel values above the threshold value have been set to one while all the others to zero. The image has been scaled to 16 × 16 pixels as μBrain only supports up to 256 input channels. We show samples from the dataset in the right panel of Figure 9.

As per the MNIST use case, we have trained a traditional ANN, and then we have converted it into a spiking neural network. The binary images [0,1] have been mapped with input frequencies equal to 0 Hz and 655 kHz. As previously, we have evaluated the output of the network using a single measure of ISI. The output neuron index with the lowest ISI predicts the input class. Using this dataset, we have achieved an accuracy of 93.4% and energy consumption of 340 nJ per classification. Table 1 show the confusion matrix for the radar-gesture classification on the test set.

**Table 1:** The confusion matrix for on-chip classification of the radar gesture dataset (test-set).

	**Hello**	**Toward**	**Horizontal**	**Background**
Hello	70	0	0	5
Toward	0	66	5	4
Horizontal	0	6	120	0
Background	2	0	0	55

For comparison, in Scherer et al. (2020), the authors developed a very low power embedded processing system for real-time gesture recognition based on radar sensing, which achieves 86.6–92.4% accuracy with energy consumption per classification of 4.52 mJ on inputs from a constellation of high-resolution 60 GHz FMCW radars. One of the two datasets they consider (11-gesture) includes fine gestures with fingers, while the other one (5-gesture) contained more coarse-grained gestures analogous to ours. The radar sensor we used is a much lower resolution (operating at only 8 GHz, with a range resolution in the order of ten of cm instead of sub-cm), and the antenna we used does not provide angular information therefore, the samples are much less informative. The networks they trained were one 2D-CNN (seven layers deep) in tandem with a 1D TCN (10 layers deep) with 16 bit fixed-precision weights, which is to be contrasted with our 2–3 layer SNN of only 4-bit weight precision. Nevertheless, the accuracy we achieve is competitive while our energy consumption per classification is 3-plus orders of magnitude lower, making our solution truly an ultra-low-power one.

While not directly comparable (but rather as an indicative reference), this performance is on par with results in the literature based on the DvsGesture dataset (Amir et al., 2017) for gesture recognition from a dynamic vision sensor (Delbrück et al., 2010). Using various spiking networks and other machine learning models, the reported accuracy (Amir et al., 2017; Shrestha and Orchard, 2018; Ghosh et al., 2019; Wang et al., 2019; Kaiser et al., 2020; Maro et al., 2020) lies in the range between ~91 and 96% for 10-gesture classification. In a more closely related to our setup, the authors in Maro et al. (2020) report ~82 and ~93% accuracy with and without, respectively dynamic background suppression filtering, using a two layer network and based on a new dynamic vision sensor dataset (NavGesture) that contains five gestures very similar to ours. Last but not least, it is worth pointing that in Amir et al. (2017) from the above list, a 3,951-neuron spiking CNN was deployed in a single True North IC, measuring 44.5 mW power consumption (without the leak) for this task.

## 4. Discussion

This paper introduced μBrain, a lightweight neuromorphic inference engine for ultra-low power applications in the IoT domain. It offers an alternative to neural network accelerators when there is a high degree of sparsity (temporal, low-rate) in the input signal that can be exploited to reduce power consumption. Off-the-shelf deep-learning accelerators for edge inference, such as Google EdgeTPU (Cass, 2019), Intel Movidius (Ionica and Gregg, 2015), and Nvidia Jetson (Mittal, 2019) perform a competitive number of operations per watt. However, they cannot efficiently exploit sparsity in the signals to scale their energy use. This means that when the input signal is highly sparse (e.g., natural signals like audio/video/EEG/etc.), they end up performing a large number of redundant operations, which can be skipped. For example, when the sparsity is higher than 95%, <5% of operations are required, and the remaining are just overhead. In deep learning algorithms achieving over 70% activation sparsity while maintaining accuracy within 2% is challenging (Wen et al., 2016; Kurtz et al., 2020). By contrast, in Yin et al. (2020) SNN architectures achieve a very high degree of spatio-temporal sparsity (more than 95%) with negligible accuracy loss.

Compared to many typical ANN accelerators for edge AI, μBrain inherently exploits all types of sparsity (spatial, structural, and temporal) in achieving its ultra-low-power signal processing tasks. Spatial and temporal sparsity relate to neuron activations, while structural sparsity relates to synaptic weights. μBrain takes advantage of spatial sparsity by operating in a truly event-driven fashion: computations take place only for the parts of the input that are non-zero and only when a non-zero activation is propagated through the network, all other lateral parts of the network remain silent conserving energy. It also takes advantage of temporal sparsity since it uses stateful neurons: the memory potential in each neuron is integrating the changes of its inputs, state is thus updated only when there are changes between subsequent inputs and a neuron fires and activates other down-stream neurons only when there is sufficient amount of change in the inputs (level crossing). In the absence of any input spikes nothing is active downstream (conserving energy) until there is a change (spike) in space or time. Finally, structural sparsity is programmable in μBrain at synthesis time. Suppose a model has a pruned network topology. In that case, μBrain can be synthesized with reduced synaptic connectivity, which saves area and static power for maintaining weight memory which would otherwise be set to zero as at runtime (an overhead in fully connected crossbar architectures). To give an impression of the related energy costs and savings from reducing spike activity (dynamic power) and synaptic connectivity (static power), in the topology of the MNIST use-case (section 3.2), we measure on average 11,500 spikes per classification (for 6,400 input stimuli per image), where μBrain consumes around 26pJ per spike (including communication, neuron accumulation, and synaptic read) and out of which 30% is static power^3^. Reducing the network connectivity (structural sparsity) or increasing the speed of the network reduces linearly the static power expended due to leakage. Increasing the thresholds in the neuron parameters (spatio-temporal sparsity) also reduces the dynamic power.

One big challenge in digital neuromorphic chips and μBrain's design is static power consumption (leakage power). While the architecture is designed to have event-driven dynamic power consumption (consume dynamic power only when there is an event), there is no control on static power. Since the architecture area is dominated by memory, most of the static power is consumed to keep the flip-flop-based memories alive. However, this challenge can be tackled at various levels, such as using Fully-Depleted Silicon-On-Insulator (FDSOI) (Carter et al., 2016) manufacturing technology, advanced non-volatile memory technologies (Burr et al., 2017), digital design tricks (e.g., power gating when no inputs are present), and by pruning at synthesis time unneeded synaptic connectivity (as discussed above).

μBrain has been designed to offer flexibility and customizability for different applications in the IoT domain. This means that it is possible to change the number of neurons in each layer, the number of layers, connectivity structure, and the parameters' resolution. The design incentive is to empower in this way IoT applications where power consumption is the number one priority and make integration with various sensors effortless (more often than not by packaging μBrain and the sensor in the same IC); to perform tiny machine learning tasks that were not possible or affordable (energy-wise) before. It is less efficient for implementing very deep neural networks as silicon area efficiency plays an essential role. The lack of time-multiplexed neuron cores in μBrain limits the scalability. However, avoiding time-multiplexing of neuron processing has been a conscious trade-off given the target application domain (i.e., small networks, energy efficiency), since it has enabled the co-location of memory and processing.

Another aspect that, at first sight, might appear as a limitation of μBrain is the use of Integrate-and-Fire (IF) neurons. However, there is recurrent synaptic connectivity among neurons the absence of leakage in the neurons may see as unnecessarily restrictive to the effectiveness of recurrent network architectures. In practice, however, quite the opposite holds. It is easy to introduce leakage at a fine-grained neuron level (different leak functions and with varying parameters per neuron); by sacrificing for this purpose, one neuron's inputs. This choice has been motivated by the intended use of μBrain primarily for experimental purposes.

Finally, one current inconvenience in the μBrain architecture is that the delay cell, which is one of the critical components, requires re-customization when ported to different manufacturing technologies. Moreover, while there is an advantage in going to small node technologies in terms of power consumption and area, the delay cell's speed will remain the same in practice. While this is a minor nuisance, it is slightly at odds with the otherwise general design portability provided by the synthesizability in a complete digital design.

### 4.1. μBrain and Low-Power Neuromorphic Devices

Several other ultra-low-power neuromorphic processors have recently been developed. Table 2 compares our proposed architecture with the other state-of-the-art neuromorphic architectures for which the power consumption reported is <120 mW. Among them, μBrain achieves competitive energy consumption per prediction (308 nJ/MNIST classification) without compromising accuracy. It is an entirely event-driven design (i.e., consumes only leakage power in the absence of input) and is fully synthesizable.

**Table 2:** Reference comparison of μBrain with other neuromorphic processors for the MNIST handwrittend digit classification.

	**μBrain**	**Frenkel et al. (2018)**	**Park et al. (2019)**	**Cho et al. (2019)**	**Chen et al. (2018)**	**Moradi et al. (2017)**	**Davies et al. (2018)**
MNIST accuracy (%)	91.7 (16 × 16)	91.4 (16 × 16)	97.83	91.6 (16 × 16)	97.9	–	96.4
Neuron/Synapses used for MNIST	74/17k	10/2.5k	410/199k	2048/149k	1546/666k	–	10/7840
VDD (V)	1.1	0.55–1.1	0.8	0.7	0.525–0.9	1.3–1.8	0.5–1.25
Energy/Prediction (nJ)	308	15 @ 75 MHz, 54 @ 1.3 MHz	236.5	–	1700	–	85,52^*^
Technology (nm)	40	28 FDSOI	65	40	10 FinFET	180	14 FinFET
Physical neurons cores/total neurons	336/336	1/256	410/410	2048/2048	4096/4096	1024/1024	128/131072
Power	73 μW	35–447 μW	23.6 mW	46.6 mW (2.3 uW * 4096 neurons)	94 mW	400 μW @ 10 Hz average firing rate	110 mW
Area (mm^2^)	2.68 (1.42 core only)	0.086^**^	10.08	2.56	1.7	43.79	60
Synaptic resolution # bits	4	4	>10	2/3	7	2 (analog)	1–9
Clock frequency	Event-driven	75 MHz	20 MHz	Global Async. Locally sync 110 MHz (neurons)	105 MHz	Event-driven	Event-driven
Fully synthesizable	Yes	Yes	Yes	Yes	Yes	No (Analog Mixed Signal design)	Yes
Supported algorithm	SNN feed-forward, recurrent	SNN online learning, feed-forward	SNN on-line learning	SNN feed-forward, recurrent	SNN/BNN online-learning, feed forward, recurrent	SNN feed-forward, recurrent	SNN, online-learning, feed-forward, recurrent

*The μBrain's power is measured with the input frequencies of [100, 655 kHz], this result in an average time per classification of 4.2ms*.

* *Blouw et al. (2019)*.

** *Only IP core area without peripheral and pads*.

μBrain should be categorized as a small-scale neuromorphic processor. Unlike large-scale processors (like Davies et al., 2018), where the power consumption is several *m*W, small-scale processing units like μBrain only consume a few μW and therefore can be integrated with battery-powered always-on devices (for example, in wearable or implantable devices). Additionally, these processors can be integrated with the sensors to build a highly efficient sensor-processor system-on-chip (SoC).

Frenkel et al. (2018) designed and implemented a 256-neuron processor with online learning capability and time-multiplexing of an entire topology in a single physical neuron core. The neurons in this design are fully connected (256 × 256 synapse), which allows for arbitrary topologies. However, this high amount of synaptic connections is an overhead not required for many applications. In μBrain, our approach is to sacrify runtime flexibility for efficiency. Therefore, we decided to perform mapping-synthesis co-optimization. After synthesis and fabrication of the chip, in μBrain, it is only possible to modify the synaptic weights of the SNN but not the main configuration (synaptic connectivity). This saves substantial area and allows for highly efficient implementation of the processing unit for a target application (for example, when integrating with a radar sensor).

Also, by contrast to Frenkel et al. (2018) as well as Davies et al. (2018), μBrain does not time-multiplex neurons in neuron cores, which leverages the co-localization of memory and compute (to improve latency and energy consumption).

Park et al. (2019) also presented a clocked SNN architecture processor, but the proposed processor consumes over 20 mW and cannot be used for always-on, battery-powered applications. In contrast to this work and Frenkel et al. (2018), μBrain does not use a fixed clock frequency, making it more efficient for event-based applications. Compared to other event-driven ASICs like Davies et al. (2018), the shallow processing pipeline of μBrain allows for a lightweight oscillator to generate just a few pulses upon each event's arrival.

Moradi et al. (2017) presented an analog neuromorphic processor. Even though the analog design has clear advantages over the digital one, it is not easily integratable and synthesizable with other digital units (e.g., sensors) and therefore different from our proposed solution. As we discussed before, analog design is also vulnerable to manufacturing variations, making its simulation and training in software difficult. It is challenging to use for critical applications like healthcare. Nevertheless, μBrain gets as close as possible to an analog design by featuring a clock-less architecture (truly event-driven) and co-localizing computation and memory in the same die.

## Data Availability Statement

The datasets presented in this study can be found in online repositories. The names of the repository/repositories and accession number(s) can be found at: https://github.com/federicohyo/8GhzGestureDataset.

## Author Contributions

JS and FC designed the μBrain architecture and performed the experiment. JS implemented the μBrain's architecture in digital logic. FC and MS collected the dataset and performed the pre-processing. FC designed the experiment. All authors contributed with discussions and assisted in editing the manuscript.

## Conflict of Interest

The authors declare that the research was conducted in the absence of any commercial or financial relationships that could be construed as a potential conflict of interest.

## Footnotes

^1^In this respect, input spike arbitration does not preserve the timing of inter-arrivals.

^2^Note that the actual mean rate frequencies are not significant: it is their frequency ratios that matter.

^3^These numbers are for Vdd 1.1 V in 40 nm technology, with 53 μA leak current and 74 μA total current in 42 s of classifying 10,000 samples.
