# Supplementary material for: μBrain: An Event-Driven and Fully Synthesizable Architecture for Spiking Neural Networks
Source: Front Neurosci. 2021 May 19;15:664208. doi: 10.3389/fnins.2021.664208 (PMC8170091; doi:10.3389/fnins.2021.664208)
Supplement: Supplementary file 1 [file Data_Sheet_1.PDF]

Supplementary Material

1 SUPPLEMENTARY MATERIAL

1.1 micro-Doppler radar processing

To compute the micro-Doppler maps, we calculate the vector $R_n[b]$ for $n = 1, ..., N_{chirps} *_N frames$, where b is the range-bin in which the gesture is performed (i.e. corresponding to two meters distance), N_{chirps} is the number of chirps in a frame, and N_{frames} is the number of frames considered in a gesture ($N_{chirps} = 192$, $N_{frames} = 3$ in this work). We compute $R_n[b]$ as follow

$$R_n[b] = \frac{1}{\sqrt{L}} \sum_{i=0}^{L} w_b[i] c_n[i] e^{-j2\pi \frac{ki}{L}} \tag{S1}$$

where $L = 512$ is the number of ADC samples of on chirp c_n (the received IF signal for chirp number n) and w_b denotes the Blackman window.

Then, by applying the Short-Time Fourier Transform (STFT) on $R_n[b]$ (Allen (1977)):

$$\Theta[m, f] = \sum_{n=-\infty}^{\infty} R_n[b] H_s[n - mR] e^{-j2\pi fn} \tag{S2}$$

where H_s is the Hanning window of length s, (defining the time-frequency resolution of the transform), and R is the hop size ($s = 192$ and $R = 8$). The micro-Doppler signature plot is found by taking the magnitude of $\Theta[m, f]$ (Wu et al. (2018)). The size of the $\Theta[m, f]$ matrix for each acquisition of is $(N_T \times s)$ with $s = 192$ frequency bins and the number of time bins N_T given by Banerjee et al. (2020):

$$N_T = \left\lfloor \frac{N_{frames} N_{chirps} - N_{overlap}}{s - N_{overlap}} \right\rfloor \tag{S3}$$

where $N_{overlap}$ is the number of overlapping bins between subsequent analysis windows and is equal to $s - R$.

In order to obtain example patches to feed to our neural network, we cut $|\Theta[m, f]|$ along dimension m into patches of 714 ms worth of time.

1.2 Hand-gesture 8GHz radar dataset

We distribute the dataset containing the pre-processed radar data at this location https://github.com/Comp4Drones

1.3 μBrain scaling

Scalability to the level of accommodating very deep network topologies is outside the scope and mission of μBrain as its target is IoT applications with low-data rate, and battery powered systems. However, here we provide estimates on some hypothetical scaled-up versions of the IC.

These estimations are carried for some reference network architectures. In the following table, INP refers to the number of inputs (Address Event Represented on the Input interface). RNN represents a recurrent layer, FF represents the number of neurons in a feed-forward layer. Among consecutive layers, we assume

full connectivity. The lateral connections for the RNN layers is kept to 30$\%$ as in the prototype device that has been fabricated. These estimates are carried using the following assumptions (parameters of the architecture):

- Technology: TSMC CLN40LP (40nm CMOS)
- Neuron accumulator size: 7 bits
- INP connections to only one individual neuron of the first RNN layer
- RNN lateral connectivity 30$\%$
- Arbiter spike buffer 1-bit

We have estimated assuming linear scaling with the static power compared to the implemented μBrain chip (scales linearly with the area). The total power is the sum of the static power and the dynamic power. The dynamic power is estimated assuming 10$\%$ synaptic activity at the cost of 9nJ per synaptic event (including synapse read, accumulation, and communication, as it is for the μBrain IC. As a reference, the μBrain device has a core area of $1.42mm^2$, static power consumption of 54 μW, and a dynamic power consumption per spike of about 9 nJ. This makes its dynamic power consumption equal to its number of synapses (19878 * 10%) $\sim 20\mu$W, which means a total power consumption of about 74 μW.

**network**	**synapses**	**neurons**	**core area** (mm^2)	**power** (μ**W**)
INP 256, RNN 256, FF 64, FF 16	19878	336	1.42	74*
INP 256, RNN 256, RNN 256, FF 64, FF 16	19481	592	2.21	223
INP 256, FF 256, FF 256, FF 16	135952	528	4.97	320
INP 256, RNN 512, FF 128, FF 32	201785	672	5.05	402
INP 256, RNN 1024, FF 256, FF 64	542579	1344	21.56	1351

*Measured from the IC prototype

As it can be seen from table1.3, and explained in the manuscript, the number of synapses dominates the area of the devices. In a scaled-up version the problem of memory density will need to be addressed.

REFERENCES

Allen, J. (1977). Short term spectral analysis, synthesis, and modification by discrete fourier transform. *IEEE Transactions on Acoustics, Speech, and Signal Processing* 25, 235–238

Banerjee, D., Rani, S., George, A. M., Chowdhury, A., Dey, S., Mukherjee, A., et al. (2020). Application of spiking neural networks for action recognition from radar data. In *2020 International Joint Conference on Neural Networks (IJCNN)* (IEEE), 1–10

Wu, Q., Zhao, D., et al. (2018). Dynamic hand gesture recognition using fmcw radar sensor for driving assistance. In *2018 10th International Conference on Wireless Communications and Signal Processing (WCSP)* (IEEE), 1–6
